# Community assembly history alters relationships between biodiversity and ecosystem functions during restoration

**DOI:** 10.1002/ecy.3910

**Published:** 2022-12-21

**Authors:** Christopher P. Catano, Anna M. Groves, Lars A. Brudvig

**Affiliations:** ^1^ Department of Plant Biology Michigan State University East Lansing Michigan USA; ^2^ Program in Ecology, Evolution, and Behavior Michigan State University East Lansing Michigan USA; ^3^ Freelance Science Journalist Kansas City Missouri USA

**Keywords:** alternative states, colonization, decomposition, floral resources, functional traits, historical contingency, primary productivity, seed dispersal, stochasticity, succession, tallgrass prairie, year effects

## Abstract

Relationships between biodiversity and ecosystem functioning depend on the processes structuring community assembly. However, predicting biodiversity‐ecosystem functioning (BEF) relationships based on community assembly remains challenging because assembly outcomes are often contingent on history and the consequences of history for ecosystem functions are poorly understood. In a grassland restoration experiment, we isolated the role of history for the relationships between plant biodiversity and multiple ecosystem functions by initiating assembly in three different years, while controlling for all other aspects of community assembly. We found that two aspects of assembly history—establishment year and succession—altered species and trait community trajectories, which in turn altered net primary productivity, decomposition rates, and floral resources. Moreover, history altered BEF relationships (which ranged from positive to negative), both within and across functions, by modifying the causal pathways linking species identity, traits, diversity, and ecosystem functions. Our results show that the interplay of deterministic succession and environmental stochasticity during establishment mediate historical contingencies that cause variation in biodiversity and ecosystem functions, even under otherwise identical assembly conditions. An explicit attention to history is needed to understand why biodiversity‐ecosystem function relationships vary in natural ecosystems: a critical question at the intersection of fundamental theory and applications to environmental change biology and ecosystem restoration.

## INTRODUCTION

Over two decades of research and hundreds of experiments show that biodiversity influences ecosystem functioning, for example, carbon dynamics and nutrient cycling (Cardinale et al., 2011; O'Connor et al., 2017). As a result, global sustainability initiatives, such as the Intergovernmental Panel for Biodiversity and Ecosystem Services and the United Nation's Decade on Ecosystem Restoration, make biodiversity a focal point for maintaining ecosystems' contributions to people (Isbell et al., 2017). However, biodiversity‐ecosystem functioning (BEF) relationships in nature are complex and global syntheses show that BEF relationships can vary in both strength (slope) and direction (from positive to negative) (Cardinale et al., 2012). Understanding why BEF relationships vary in natural settings is critical to advance BEF theory and to develop capacities to predict ecosystem responses to biodiversity change.

Variation in BEF relationships can depend on how underlying community assembly processes, like dispersal and species sorting across environmental gradients, structure community composition and its consequences for ecosystems (Leibold et al., 2017; Mori et al., 2018). Yet, natural assembly processes are often prevented in BEF experiments that limit environmental variation or prevent colonization/extinction dynamics, for example, by weeding out non‐seeded species in plant BEF experiments (Tilman et al., 1996). Therefore, the processes that cause positive BEF relationships in experiments—complementarity and selection effects (Loreau et al., 2001)—can cause different relationships between ecosystem functions and the realized diversity shaped by community assembly (Hagan et al., 2021). However, linking community assembly to variation in BEF relationships is challenging because biodiversity outcomes are often contingent on history (chance events early in assembly that lead to alternative states or successional trajectories) (Fukami, 2015), and the consequences of historical contingencies for emergent ecosystem functions are rarely quantified.

Community assembly history reflects differences in immigration, variation in initial environmental conditions that influence establishment, and the resulting successional dynamics. To date, though, the few studies that evaluated the consequences of history on biodiversity and ecosystem functioning focused mostly on biotic contingencies (but see Manning & Baer, 2018), particularly differences in species arrival order that caused priority effects. For example, Fukami et al. (2010) and Dickie et al. (2012) showed that immigration history in fungal decomposer communities altered species diversity and carbon dynamics. Similarly, Delory et al. (2019) showed that priority effects in a grassland experiment altered the mechanisms underpinning plant biomass overyielding. These studies demonstrated that immigration history influenced species composition and ecosystem functions individually; however, neither study quantified how history altered the nature (strength or direction) of BEF relationships. Therefore, the importance of history for contributing to variation in BEF relationships remains unknown.

Moreover, studies have not considered how abiotic history, such as temporal variation in disturbance and resource pulses (Holt, 2008), alter BEF relationships. For example, year effects—interannual variation in weather, consumer abundances, and/or pathogens (Werner et al., 2020)—can cause historical contingencies in community assembly even when environmental conditions and immigration sequences are identical (Groves et al., 2020; MacDougall et al., 2008; Stuble et al., 2017). Year effects thus represent a form of environmental stochasticity that modifies the initial conditions that influence colonization from the regional pool. Whether year effects alter ecosystem functioning will depend on the extent to which functionally different species establish and whether these differences persist or give way to convergent successional dynamics (Manning & Baer, 2018). Despite the increasingly clear role of year effects for causing variation in community assembly, studies have not quantified their consequences on BEF relationships. Studies are needed that replicate community assembly across years in the same environments to evaluate if repeatable BEF relationships emerge (e.g., due to deterministic succession). If replicated assembly does not lead to similar BEF relationships, then year effects due to stochastic elements of history (chance establishment, environmental stochasticity, ecological drift) could render BEF outcomes difficult to predict regardless of similar immigration history.

In a grassland restoration experiment, we evaluate how assembly history mediated by year effects altered community structure, and the consequences for variation in BEF relationships. To isolate the role of year effects, we manipulated the year of seed arrival, while controlling for other aspects of community assembly (species pool size and composition, seed arrival rate and sequence, soil resources, and landscape context). This experimental design helps overcome difficulties of observational studies where year effects are confounded with differences in species pools and environmental conditions across sites and provides one of the first experimental evaluations of year effects as a mechanism generating variation in BEF relationships. We then followed species and trait community trajectories over 6 years. In the final 2 years, we quantified biodiversity and three ecosystem functions: two related to carbon dynamics (net primary productivity and decomposition rate) and a third related to trophic dynamics (floral resource production). We asked three overarching questions:

1. Does establishment year cause variation in plant species and trait community structure?
2. What are the consequences of establishment year on multiple ecosystem functions and their relationships (strength and direction) with biodiversity?
3. To what extent is variation in BEF relationships related to deterministic succession versus stochastic year effects?

We hypothesized that variable BEF relationships would emerge if year effects, for example due to differences in weather during seed arrival, altered species and functional trait trajectories. In contrast, similar BEF relationships would emerge due to succession if communities established in different years did not vary in their trait trajectories, for example, if environmental filtering due to soil resources selects for functionally similar communities regardless of initial conditions. By using experimental restoration as a model system, this study is uniquely positioned to advance BEF theory in complex, natural ecosystems and provide practical solutions to ecosystem restoration.

## METHODS

### Overview and experimental design

We experimentally manipulated establishment year through ecological restoration of tallgrass prairies at Michigan State University's W. K. Kellogg Biological Station—Lux Arbor Reserve in Michigan, USA. To control for differences in environmental conditions across plots, we selected a contiguous area where all plots share the same land‐use history, landscape context, and soils. The plant community prior to our experiment was typical of old‐field ecosystems in this region; dominated by *Bromus inermis* (Smooth brome), *Centaurea stoebe* (Spotted knapweed), and a mix of other weedy perennials.

We divided the area into 18,500‐m^2^ plots, and randomly assigned each plot to one of three establishment year treatments: 2014, 2015, or 2016. During each establishment year, we restored plots using the exact same methodology to replicate initial community assembly. First, to promote colonization from the species pool we disturbed the plots by mowing to stimulate growth, applying glyphosate herbicide to remove old‐field vegetation, and tilling the soils. Second, we constructed a native prairie species pool using eight species spanning diverse functional groups including C_3_ and C_4_ grasses, legumes, and non‐leguminous forbs (Appendix S1: Table S1). We manipulated immigration from the pool into each plot by hand broadcasting seeds at a constant rate of 379 live seeds/m^2^ with the same number of live seeds per species, then used a cultipacker to increase soil‐seed contact and germination. To minimize seed source variability, we acquired all seeds from the same native seed vendor (Native Connections; Three Rivers, MI, USA), who acquired seeds from the same regional seed producers each year. We also ensured each establishment year treatment received the same number of live seeds by adjusting mixes based on viability tests performed by the seed supplier each year. Third, each plot was managed identically and sequentially with respect to its establishment year: mowed annually in the first 2 years to help seeded species establish and prescribed burns (hand‐ignited backing fires) in the spring prior to the fourth growing season to mimic natural fire regimes that maintain native tallgrass prairies. Therefore, all aspects of community assembly (species pool size and composition, immigration rate, environmental conditions, and disturbance) were standardized across plots, leaving the year in which community assembly was initiated (i.e., establishment year) as the only variable (see Groves & Brudvig, 2019 for full experimental details).

### Quantifying species and trait diversity

In 2019 and 2020, we identified the percent cover of all species (visually estimated by a single observer; C.P.C.) in 1‐m^2^ subplots spaced 5‐m apart along transects in each of the 18 plots. The 2020 transects were offset by 10‐m to avoid potential effects of the prior year's sampling (e.g., trampling) and to avoid plots from other studies within this experiment. Therefore, each establishment year had six 500‐m^2^ plots, each with eight 1‐m^2^ subplots (four sampled in 2019, four sampled in 2020; total *n* = 144). We identified 69 species across the experiment, then quantified species diversity in each subplot as Shannon Diversity (exponential of Shannon entropy) and differences in species composition with Bray–Curtis dissimilarity; both calculated with vegan v2.5‐7 (Oksanen et al., 2020).

To quantify trait composition and diversity, we measured leaf, seed, and whole plant traits associated with variation in plant life‐history strategies and functions. We measured three quantitative traits—plant height (*H*_max_), specific leaf area (SLA), and seed mass—for each of the 26 most common species that represented over 95% of the total species cover in each subplot. Traits were measured on healthy, flowering individuals from this experiment and from nearby restored tallgrass prairies in similar environments (Zirbel et al., 2017). *H*_max_, mean distance from the ground to the highest photosynthetic tissue averaged across 20 individuals per species, is associated with fitness differences in response to competition for light, dispersal distance, and reproductive ability between disturbances (Moles et al., 2009). SLA, leaf area per unit dry mass averaged from two leaves from each of 10 individuals, is correlated with relative growth rates across gradients of light and soil resource availability (Wright et al., 2004). Seed mass, mean dry mass of 50 seeds for each species, is correlated with dispersal and establishment ability (Westoby, 1998). We averaged trait values across individuals to estimate population means for each species. Intraspecific trait variation is likely to be small in our study because we sampled all individuals from the same populations and in similar environments. We square‐root transformed seed mass and *H*_max_ to normalize trait distributions for analysis. We quantified trait diversity for each subplot using functional dispersion (which is independent of species richness), calculated with FD v1.0‐12 (Laliberté et al., 2014), and quantified differences in trait composition across subplots as the Euclidean distance among community weighted trait means.

### Quantifying ecosystem functions

We quantified three ecosystem functions in 2019 and 2020 for each of the 144 subplots distributed across the three establishment years: above‐ground net primary productivity (ANPP), decomposition rate, and floral resource production. We measured ANPP by clipping all plant biomass from a 0.5‐m^2^ section of the subplots at peak biomass (late July). We removed dead plant material from prior year's growth and quantified biomass produced over the growing season (g m^−2^) after drying the biomass for 48 h at 65°C. We measured decomposition rate as the mass lost from a standard substrate (3.80 ± 0.01 g of cellulosic fiber paper) stapled to the soil surface in each subplot at the start of the growing season (May). Using a standard substrate relates decomposition to differences in local biotic (e.g., microbial composition) and abiotic environment (e.g., temperature), rather than differences in plant tissue composition. We sealed the fiber paper inside of 15.5 × 15.5‐cm mesh bags (mesh size 2 mm^2^) to prevent loss due to small mammals or other physical disturbance. We collected the fiber paper after 161 days spanning the growing season and prior to the first frost, dried them at 65°C for 48 h, then calculated decomposition rate as (starting dry mass − final dry mass)/days deployed. We measured floral resource production as the total cover of animal‐pollinated flowers in each subplot, summed across three sampling periods stratifying times when most species flower (June, July, and August). Floral resource production is critical for energy transfer across trophic levels and for sustaining pollinators that provide crop pollination services (Blaauw & Isaacs, 2014).

### Analysis

To determine how assembly history altered species and trait community structure, we first quantified species and trait composition trajectories over 6 years for communities assembling in each establishment year using non‐metric multidimensional scaling. We tested for differences in composition among establishment years and through time using permutational multivariate analysis of variance (McArdle & Anderson, 2001). To quantify trajectories, we included species cover data surveyed in the first 2 years of the experiment (2015 and 2016). These surveys were identical in methodology, except three 1‐m^2^ subplots per plot were surveyed in each year instead of four subplots per year as in 2019 and 2020. Therefore, species and trait composition trajectories spanned 5 years for the 2016 establishment year plots (sampled in 2016, 2019, and 2020) and spanned 6 years in the 2015 and 2014 establishment year plots (each sampled in 2015, 2016, 2019, and 2020). We quantified marginal effects of establishment year (factor) and plot age (continuous numeric) in additive models to determine the unique contributions of year effects and succession for causing variation in community composition.

To quantify how establishment year alters species diversity, functional dispersion, and ecosystem functions in 2019 and 2020, we fit linear mixed models with lme4 v1.1‐27.1 (Bates et al., 2015) using Restricted Maximum Likelihood and Kenward‐Rogers estimated denominator degrees of freedom. We coded “plot” as a random effect to account for the hierarchical design with 1‐m^2^ subplots nested within 500‐m^2^ plots. Because plots were sampled over 2 years (72 in 2019 and a different 72 in 2020), we also quantified differences in effects across these sampling periods. In all cases the effect of establishment year on biodiversity and ecosystem functions did not statistically differ across sampling years (Appendix S1: Figure S1 **)**, so we included all eight subplots for each plot in a single model. Next, we quantified how biodiversity‐ecosystem function relationships varied across establishment year treatments using the same linear mixed effects model framework described above. In these models, we included an interaction between establishment year and species diversity to quantify the extent to which BEF relationships vary; we then tested contrasts at standard plot ages and across sampling years to help disentangle the contributions of historical contingencies (via year effects) and succession for causing variation in BEF relationships (intercepts and slopes). We log_e_‐transformed ANPP and floral resource production and square‐root transformed decomposition rate to linearize relationships and ensured all model assumptions were met by inspecting residual diagnostic patterns.

Last, we developed structural equation models to gain insights into how species identities and functional traits underpin BEF relationships. We started with a model that represents links between each function and CWM trait values, functional dispersion, and species diversity. These metrics can provide insight into the roles of mass ratio effects (CWM → functions) and complementarity effects (functional dispersion → functions). We then added links from the percent cover of the dominant species in each establishment year to diversity and CWMs to test for species identity effects. We tested whether models were consistent with the data using two criteria suggested by Grace et al. (2012): χ^2^ comparing observed and saturated model *p* > 0.05 and Comparative Fit Index >0.9. Our approach was semi‐exploratory in that we tested specific cause‐effect predictions based on theory and knowledge of our system, but we considered additional links (if biologically reasonable) based on modification indices produced from residual covariances and removed links that were not statistically supported (if *p* > 0.1) (Grace et al., 2012). Last, we used partial regressions to demonstrate the relationships between all direct paths in the final model were linear. Path coefficients, uncertainties, statistical significance, and model fit were determined with global estimation using the Lavaan R package (Rosseel, 2012).

## RESULTS

**FIGURE 1:**
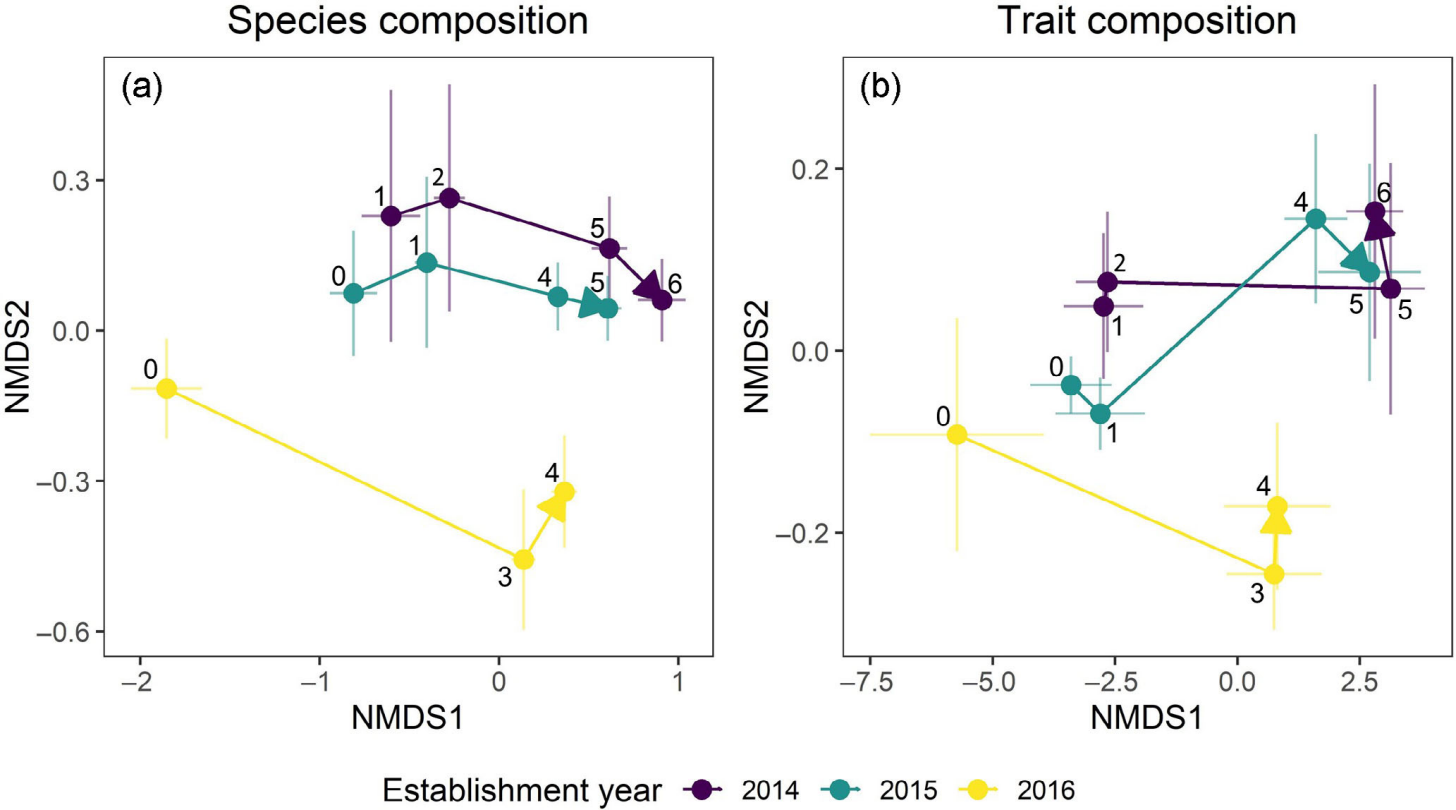
Community assembly trajectories for (a) species and (b) trait compositions in multivariate space (non‐metric multidimensional scaling). Establishment year is the year community assembly was initiated and numbers indicate the relative age of the plot in years since assembly started. Points are the centroids of community composition and error bars are 95% confidence intervals. Ordinations achieved good fit: stress values are 0.17 for species and <0.01 for traits.

Species composition differed across establishment year treatments (marginal *F* = 10.38, df = 2, *p* < 0.001) and with age (marginal *F* = 90.91, df = 1, *p* < 0.001) (Figure 1a). The communities established in 2016 started and remained on a different trajectory than those established in 2014 and 2015, which began similar to each other and appeared to be converging over time. Trait composition also differed across establishment year treatments (marginal *F* = 2.81, df = 2, *p* = 0.041) and with age (marginal *F* = 361.57, df = 1, *p* < 0.001) (Figure 1b). Mirroring species composition trajectories, the communities established in 2016 started and remained on a different trait trajectory than those established in 2014 and 2015.

**FIGURE 2:**
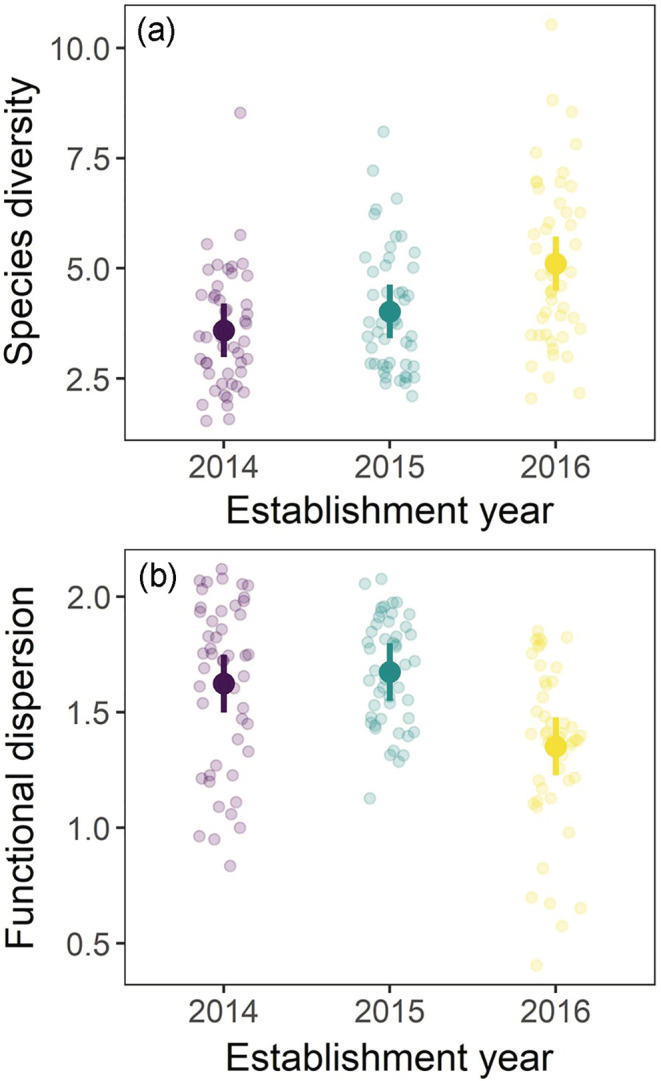
Differences in (a) species diversity and (b) functional dispersion in communities assembled from different establishment years. Points are the estimated marginal means with 95% confidence intervals from mixed effects models for diversity quantified in 2019 and 2020.

Establishment year altered both species diversity (*F*_2,15_ = 6.46, *p* = 0.009; Figure 2a) and functional dispersion (*F*_2,15_ = 7.51, *p* = 0.005; Figure 2b). Species diversity in communities established in 2016 was 27.1% greater (1.08 [−0.04, 2.22]) (mean [95% CI]) than those established in 2015 and 42.3% greater (1.51 [0.38, 2.65]) than those established in 2014; species diversity did not clearly differ between those established in 2014 and 2015 (0.42 [−0.7, 1.56]). Functional dispersion in communities established in 2016 was 19.1% lower (0.32 [0.08, 0.55]) than those established in 2015 and 16.6% lower (0.27 [0.03, 0.50]) than those established in 2014; functional dispersion did not clearly differ between those established in 2014 and 2015 (0.05 [−0.18, 0.28]).

**FIGURE 3:**
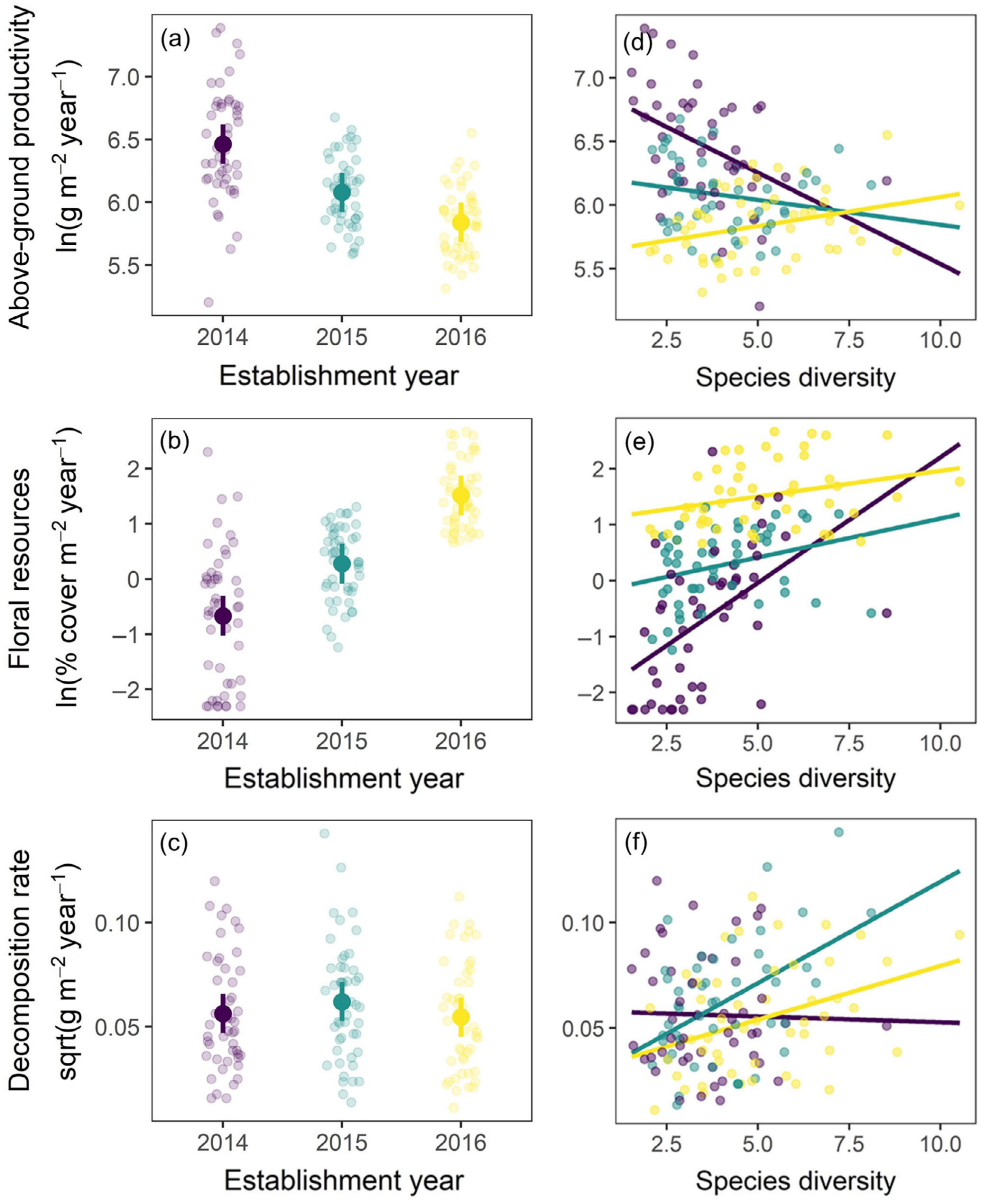
Differences in ecosystem functioning for (a) above‐ground net primary productivity, (b) floral resource production, (c) decomposition rate, and (d–f) associated biodiversity‐ecosystem functioning relationships in communities assembled from different establishment years. Panels (a–c) show the estimated marginal means with 95% confidence intervals and panels (d–f) show the predicted functions from mixed effects models.

Establishment year had varying effects on ecosystem functions (Figure 3). ANPP varied across establishment years (*F*_2,15_ = 15.87, *p* < 0.001; Figure 3a). ANPP in communities established in 2014 was 46.6% greater (204 g m^−2^ year^−1^ [45, 364]) than those established in 2015 and 86.3% greater (297 [148, 447]) greater than those established in 2016; ANPP did not clearly differ between those established in 2015 and 2016 (92 [−21, 206]). Floral resource production also varied across establishment years (*F*_2,15_ = 35.86, *p* < 0.001; Figure 3b). Floral resource production in communities established in 2016 was 263% greater (3.22% cover m^−2^ year^−1^ [0.97, 5.46]) than those established in 2015 and 975% greater (4.03 [1.86, 6.19]) than those established in 2014; floral resource production in those established in 2015 was 196.1% greater (0.81 [0.13, 1.48]) than those established in 2014. Decomposition rate did not clearly vary across establishment years (*F*_2,15_ = 0.66, *p* = 0.527; Figure 3c).

Moreover, we found BEF relationships differed across establishment year treatments and the type of ecosystem function. Biodiversity‐ANPP relationships differed across establishment year treatments (Figure 3d; interaction *F*_2,136.13_ = 9.30, *p* < 0.001, conditional/marginal *R* ^2^ = 0.53/0.43), with BEF relationships flipping from negative in the 2014 treatment (slope = −83 [−125, −41]), neutral in the 2015 treatment (slope = −16 [−46, 12]), to positive in the 2016 treatment (slope = 15 [−2, 32]). Biodiversity‐floral resource production relationships differed across establishment year treatments (Figure 3e; interaction *F*_2,137.29_ = 5.73, *p* = 0.004, *R* ^2^ = 0.64/0.60), with BEF relationships ranging from positive in the 2014 treatment (slope = 0.38 [0.11, 0.49]) to more neutral in the 2015 (slope = 0.18 [−0.06, 0.43]) and 2016 treatments (slope = 0.38 [−0.13, 0.90]). Biodiversity‐decomposition rate relationships differed across establishment year treatments (Figure 3f; interaction *F*_2,137.73_ = 3.31, *p* = 0.039, *R* ^2^ = 0.22/0.13), with BEF relationships ranging from neutral in the 2014 treatment (slope = −6.1 × 10^−5^ [−7.4 × 10^−4^, 6.1 × 10^−4^]) to positive in the 2015 (slope = 1.23 × 10^−3^ [4.5 × 10^−4^, 2.0 × 10^−4^]) and 2016 (slope = 5.1 × 10^−4^ [1.0 × 10^−4^, 9.2 × 10^−4^]) treatments. Pairwise BEF contrasts are in Appendix S1: Table S2.

**FIGURE 4:**
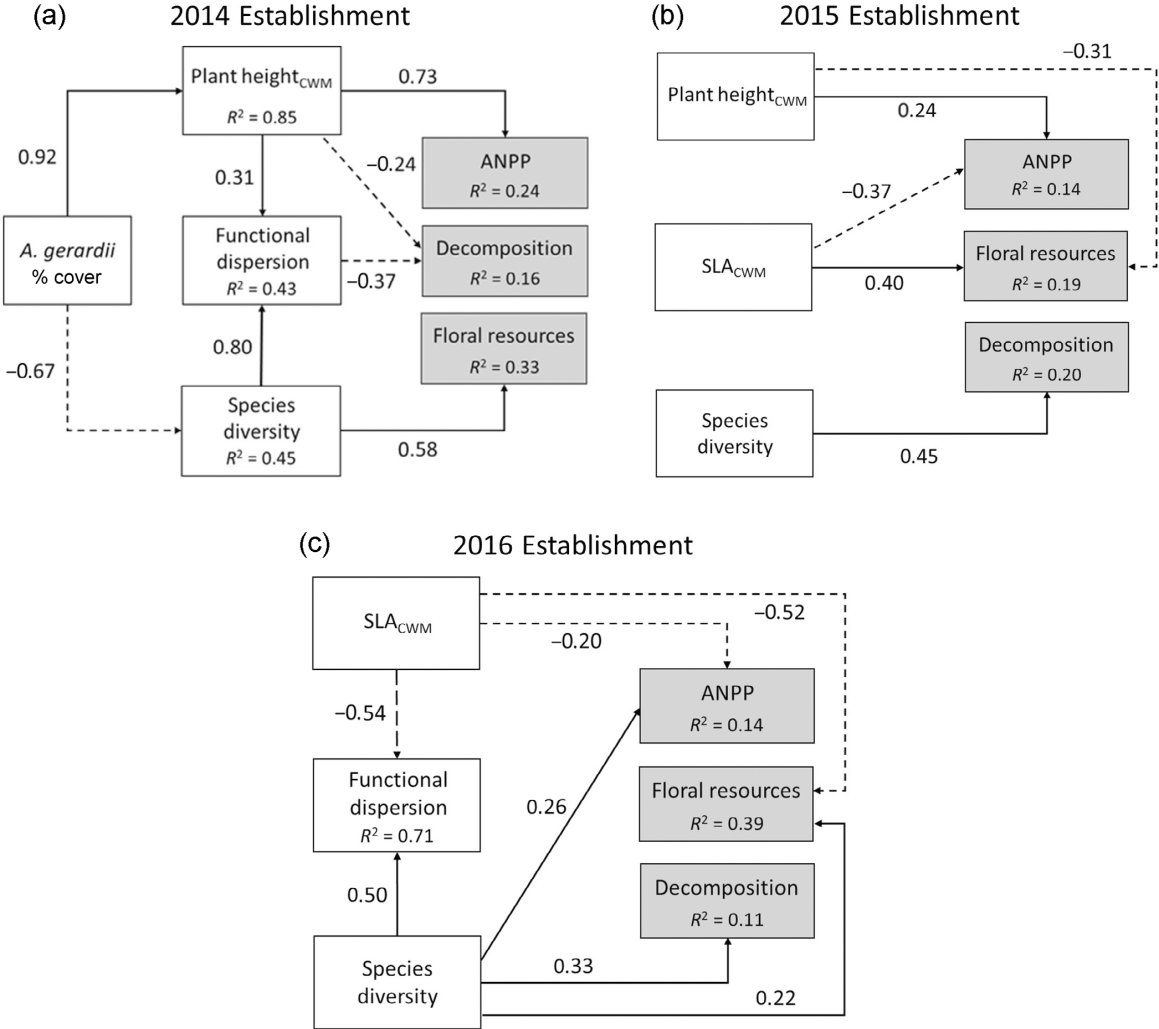
Structural equation models summarizing direct and indirect paths between species identity, community weighted mean (CWM) traits, functional dispersion, species diversity, and ecosystem functions. All paths shown (solid or dashed lines for positive or negative relationships, respectively) are the standardized path coefficients. All SEMs have close data‐model fits. 2014 establishment: *x* ^2^ = 11.16, df = 8, *p* = 0.19, Comparative Fit Index (CFI) = 0.985; 2015 establishment: *x* ^2^ = 1.97, df = 4, *p* = 0.74, CFI = 1.0; 2016 establishment: *x* ^2^ = 0.74, df = 1, *p* = 0.39, CFI = 1.0. ANPP, above‐ground net primary productivity; SLA, specific leaf area.

Last, structural equation models demonstrate direct and indirect paths between species identity, functional traits, species diversity, and ecosystem functions varied across establishment years (Figure 4; Appendix S1: Figures S2–S4). All ecosystem functions were positively associated with higher species diversity in the 2016 establishment year, whereas only decomposition was associated with diversity in the other establishment years. Instead, species identify effects and higher cover of large stature plants (higher *H*_max_) underpinned the negative diversity‐function relationships for ANPP and floral resource production. Initial SEMs were not consistent with the data, but after modifying model structures all final SEMs achieved close data‐model fits.

## DISCUSSION

While community assembly is often contingent on history (Chase, 2003; Fukami, 2015), the consequences for ecosystems remain rarely quantified. We show that different establishment years led to alternative community assembly trajectories (Figure 1) that caused variation in species and trait diversity (Figure 2). In turn, these differences altered ecosystem functions and, to some extent, their relationships with biodiversity (Figure 3). Our experiment demonstrates that under identical assembly scenarios, both historical contingencies and deterministic succession cause variation in biodiversity, carbon dynamics, and pollination resources in restored grasslands.

### The role of history in dictating community assembly

The differences in community assembly trajectories we observed can be explained by at least two, non‐mutually exclusive mechanisms: differences in successional stage and historically contingent establishment caused by year effects. First, successional stage causes variation in biodiversity when sites are at different points in their development; for example, resulting from differences in the time since colonization or disturbance (e.g., fire). Consistent with this mechanism, communities established in 2014 and 2015 are following similar trajectories where species and trait compositions are similar when aligned by age (Figure 1). Second, our results show how year effects can alter successional patterns. Communities established in 2016 started differently and are clearly following a different trajectory for both species and trait compositions. Therefore, successional stage appears responsible for differences in composition between communities established in 2014 and 2015, whereas year effects appear responsible for the persistent differences between these communities and those established in 2016.

Year effects likely result from environmental stochasticity during establishment (Werner et al., 2020). A prior study in this experiment showed variation in precipitation contributed to differences in seedling emergence and composition across establishment year treatments (Groves & Brudvig, 2019). We show here that, by 2019, communities established in 2014 and 2015 were dominated by a native C_4_ bunchgrass, *Andropogon gerardii* (big bluestem), that we seeded into the plots, whereas the communities established in 2016 were dominated by an exotic weedy forb common in the landscape, *Centaurea stoebe* (spotted knapweed) (Appendix S1: Figure S5). These results are consistent with a study of year effects in California grasslands that showed differences in precipitation and temperature during establishment led to alternative grass versus forb dominated states (Stuble et al., 2017). Year effects could also emerge from complex interactions between environmental and biotic stochasticity. For example, although we controlled immigration history by seeding all treatments with the same species at the same rates, as is typical in grassland restorations (Török et al., 2021), many species colonized from the surrounding landscape or recruited from the seedbank. These non‐seeded species dominated first‐year communities and varied in abundance across planting years due to interannual variation in weather (Groves & Brudvig, 2019), potentially resulting in priority effects that inhibited seeded species establishment in the 2016 planting year communities. It is possible that other non‐mutually exclusive factors contributed to year effects, including interannual variation in consumers or pathogens. Regardless of the exact mechanisms, our results support the hypothesis that historical contingencies due to year effects contributed to differences in community assembly that cannot be predicted by contemporary environmental conditions.

Differences in assembly trajectories between communities established in 2016 versus those established in 2014 and 2015 appear to reflect alternative states. Over the 6 years we tracked assembly, the 2016 communities have not converged towards the others (Figure 1), demonstrating persistent differences in species and trait compositions are being maintained over time. These ecosystems are managed with prescribed fire, which mimics historical disturbances that promote native species, but can cause positive frequency dependent feedbacks that promote alternative states (Crandall & Knight, 2015). Consistent with this explanation, 2021 prescribed fire temperatures were 252 and 190°C hotter on average in communities dominated by big bluestem (those established in 2014 and 2015, respectively) compared to forb dominated communities established in 2016 (Appendix S1: Figure S6). Higher fuel loads from big bluestem can feedback to maintain grass‐dominated states when hotter fires exclude more fire‐sensitive forbs. Whether differences in compositions will be stable or transient (Fukami & Nakajima, 2011) remains unknown, but so far historical contingencies in big bluestem establishment, and feedbacks with fire, appear to create alternative states through the timeframe of our study.

### The role of history in shaping BEF relationships

Community assembly history altered ecosystem functioning (Figure 3a–c), consistent with the hypothesis that history will have ecosystem‐level consequences when species differ in their traits. Moreover, assembly history altered the strength and direction of BEF relationships (Figure 3d–f), mediated through a combination of year effects and succession. Successional change (Figure 1) appears to be responsible for more negative ANPP‐diversity relationships and more positive floral resources‐diversity relationships in the 2014 compared to the 2015 establishment year communities. Consistent with this interpretation, these BEF relationships became stronger over time in these communities (Appendix S1: Figure S8 and Table S4) and were not different when compared at standardized ages (Appendix S1: Figure S7 and Table S3). Succession is resulting in increasing dominance by a native bunchgrass, big bluestem, which our SEMs show reduced species diversity while increasing ANPP, in part mediated by maximum plant height (Figure 4a) which is a trait indicative of fast growth rates and strong competitive ability (Moles et al., 2009). Lower big bluestem abundance was also associated with higher diversity of forbs, likely due to weaker competition, thus promoting a positive relationship between species diversity and floral resource production. Plant height also mediated the effect of big bluestem on decomposition rates, potentially due to shading that reduced soil moisture loss and thus promoted microbial activity. These results are consistent with a species identity effect and mass ratio theory (Grime, 1998), where the traits of the dominant species are most important for ecosystem functions. While prior studies showed biodiversity‐productivity relationships became increasingly positive over time due to stronger complementary effects (Cardinale et al., 2007), our results provide an alternative mechanism where succession resulting in increasing dominance can lead to more negative BEF relationships in naturally complex ecosystems (Appendix S1: Figure S8).

In addition to succession, historical contingencies due to year effects also appear to contribute to variation in BEF relationships. While the 2014 and 2015 establishment year communities had negative ANPP‐diversity relationships, these relationships were positive in the 2016 establishment year communities, suggesting fundamentally different mechanisms. For example, plant height was a key trait mediating BEF relationships in both the 2014 and 2015 establishment year communities (Figure 4a,b), but this trait was not important for any function in the 2016 establishment year communities. Instead, higher plant diversity and its association with better resource acquisition (higher SLA) appears to contribute to positive BEF relationships (Figure 4c). Moreover, none of the BEF relationships varied over time in the 2016 establishment years (Appendix S1: Figure S8 and Table S4), which would be expected if differences were due to succession. The 2016 establishment year communities started and remained distinct from the others in both species and trait composition (Figure 1) and were dominated by relatively low‐biomass forbs (Appendix S1: Figure S5) instead of C_4_ grasses, like big bluestem, suggesting these represent alternative states: a highly productive, native grass‐dominated state (2014 and 2015 establishment years) and a low productivity, nonnative forb‐dominated state (2016 establishment year). While evidence for year effects to alter BEF slopes was mixed, year effects lead to clear differences in average functioning when plots were at the same age, except for decomposition at 5 years (Appendix S1: Figure S7). Together, our results expand upon prior studies of history focused on species arrival order (Delory et al., 2019; Dickie et al., 2012; Fukami et al., 2010), showing how historical contingencies due to year effects and succession can contribute to variation in BEF relationships in otherwise identical environments.

### Practical implications for ecosystem restoration

Our results raise important considerations for ecosystem restoration. First, understanding how initial conditions alter restoration trajectories can help develop strategies that promote more reliable biodiversity and ecosystem‐level outcomes. Studies on year effects demonstrate that interannual variability in weather can alter plant establishment and patterns of biodiversity during restoration (Groves et al., 2020; Groves & Brudvig, 2019; MacDougall et al., 2008; Manning & Baer, 2018; Stuble et al., 2017). Our study is among the first to extend the study of year effects to BEF relationships during restoration. Such experiments help resolve variation (Brudvig et al., 2017) and inform predictive capacities in restoration (Brudvig & Catano, 2021).

We suspect historical contingencies that create alternative restoration outcomes are likely to be more consequential and long‐lasting in larger restorations. Relative to most grassland restorations, our treatment plots were relatively small (500‐m^2^) and in proximity, thereby increasing the odds that seed dispersal from nearby treatments can eventually rescue treatments that experience poor establishment (although our treatments remain distinct). Because most grassland restorations in our region are spatially separated and surrounded by other vegetation types, species that do not establish initially due to unfavorable environmental conditions have little chance of being rescued by dispersal from the surrounding area. Therefore, practitioners will likely incur costs to overseed or intervene early in restorations if year effects lead to undesirable community assembly trajectories or aspects of ecosystem functioning.

Finally, variation in biodiversity and ecosystem functioning depends on the effects of dominant species, in our case big bluestem. Big bluestem populations can quickly dominate, thereby reducing native local diversity (Grman et al., 2020) and homogenizing restorations (Catano et al., 2022). As we show here, these influences extend to aspects of ecosystem functioning and are modified by successional age and establishment year. Seeding big bluestem at lower rates could produce more successful restorations, for example by promoting higher diversity of forbs that support native pollinating insects and contribute pollination services to nearby agricultural fields (Blaauw & Isaacs, 2014). Insights into environmental conditions that lead to higher big bluestem abundance following some planting years would additionally be valuable for planning and managing prairie restoration projects.

## CONCLUSIONS

The rampant idiosyncrasies of biodiversity responses to environmental change (Lawton, 1999), ecosystem dynamics, and their emergent relationships (Cardinale et al., 2012) presents a fundamental challenge for general theory and capacities to solve pressing environmental issues. Only recently have historical perspectives gained traction to explain contingencies in biodiversity change (Fukami, 2015); however, the consequences for higher‐level ecosystem functions remains rarely quantified (Delory et al., 2019; Dickie et al., 2012; Fukami et al., 2010). We show that community assembly history shaped by succession and interannual variation in establishment conditions—year effects—can create alternative assembly trajectories that fundamentally alter relationships between biodiversity and multiple ecosystem functions. As a result, efforts to understand variation in ecosystem functioning based on contemporary environmental conditions or comparisons across sites are likely to fail in cases when history matters. While BEF studies continue to inform ecosystem management and associated policy, our research demonstrates that attention to history is needed to improve predictions on when and where biodiversity change will alter ecosystem functions and related services for humanity.

## CONFLICT OF INTEREST

The authors declare no conflicts of interest.

## Supporting information


Appendix S1

## Data Availability

Data (chcatano, 2022) are available in Zenodo at https://doi.org/10.5281/zenodo.7120895.
