# Supplementary material for: Community assembly history alters relationships between biodiversity and ecosystem functions during restoration
Source: Ecology. 2022 Dec 21;104(2):e3910. doi: 10.1002/ecy.3910 (PMC10078356; doi:10.1002/ecy.3910)
Supplement: Supplementary file 1 — Appendix S1 [file ECY-104-0-s001.pdf]

Appendix S1: Supplemental figures and tables

Christopher P. Catano, Anna M. Groves, Lars A. Brudvig

Community assembly history alters relationships between biodiversity and ecosystem functions during restoration

Ecology

Table S1: Species used to establish plots in each planting year.

**Species name**	**Common name**	**Functional group**
Andropogon gerardii	Big bluestem	C4 grass
Bouteloua curtipendula	Side oats grama	C4 grass
Elymus canadensis	Canada wild rye	C3 grass
Chamaechrista fasciculata	Partridge pea	Legume
Dalea purpurea	Purple prairie clover	Legume
Echinacea purpurea	Purple coneflower	Forb
Eryngium yuccifolium	Rattlesnake master	Forb
Penstemon digitalis	Foxglove beardtongue	Forb

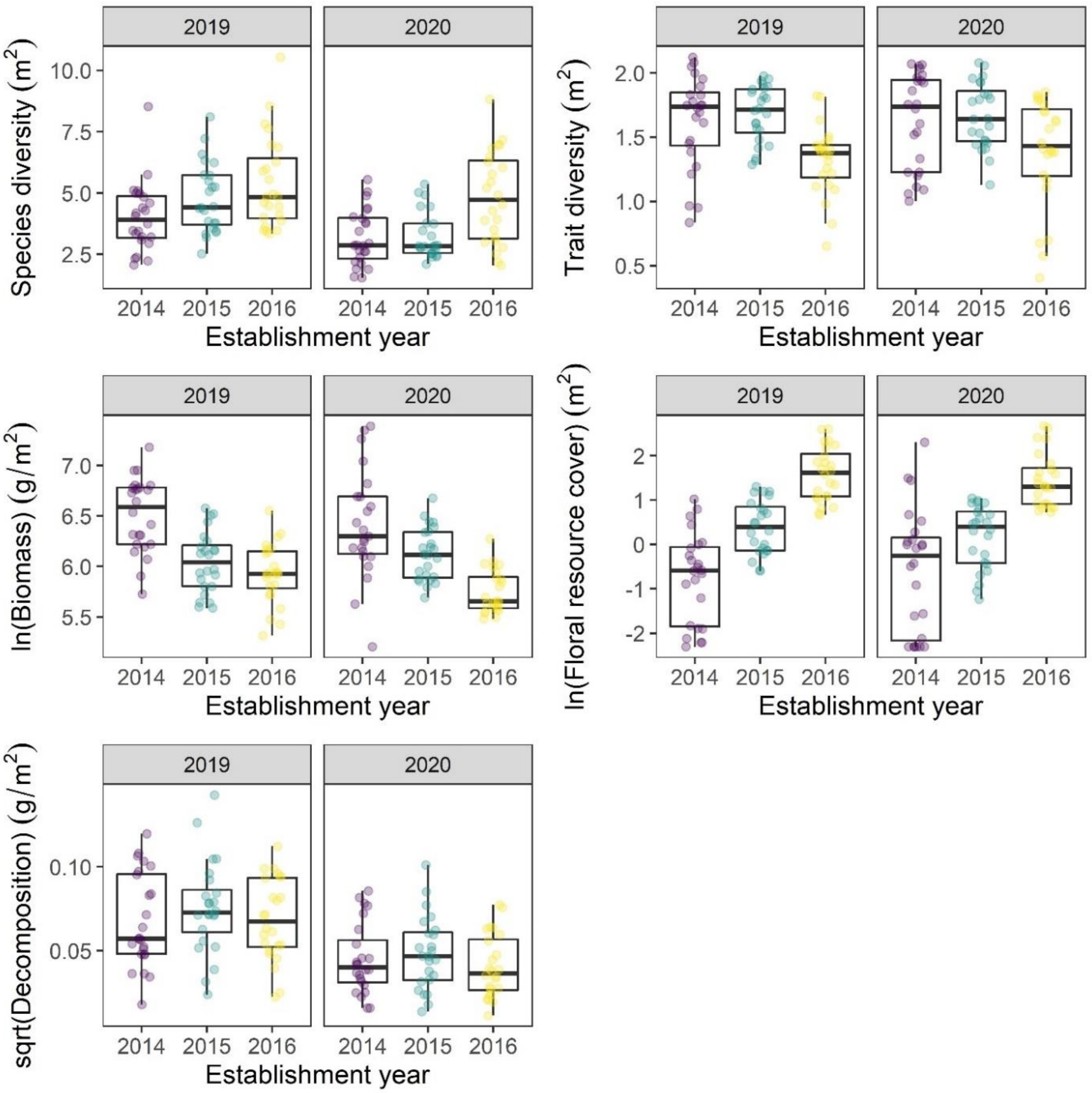

Figure S1 Effect of establishment year on diversity (species and trait) and ecosystem functions (biomass productivity, floral resource production, and decomposition rate) does not differ across sampling years (2019 and 2020). Interaction coefficient between establishment year and sampling year was not significant in all models, where each model included a random intercept for "plot"; interaction *P*-values: species diversity ($F_{2,123} = 1.65$, $P = 0.19$), trait diversity ($F_{2,123} = 0.24$, $P = 0.78$), biomass ($F_{2,122.10} = 2.20$, $P = 0.11$), floral resources ($F_{2,123} = 0.65$, $P = 0.52$), decomposition ($F_{2,123} = 0.17$, $P = 0.83$).

Table S2: Pairwise comparison of mixed model slopes between fixed effects species diversity and ecosystem functions (ANPP, Floral resource production, and decomposition rate) estimated in each establishment year. Degrees of freedom (df) were estimated with Kenward-Roger method. P-values for contrasts are adjusted for three comparisons with Tukey's method.

**Establishment year contrast**	**estimate**	**SE**	**df**	**t ratio**	**p-value**
ANPP ~ Diversity					
2014 – 2015	-0.10	0.04	136	-2.13	0.08
2014 – 2016	-0.18	0.04	137	-4.26	<0.01
2015 – 2016	-0.08	0.04	135	-2.06	0.10
Floral resources ~ Diversity					
2014 – 2015	0.30	0.12	137	2.58	0.02
2014 – 2016	0.35	0.10	137	3.30	<0.01
2015 – 2016	0.04	0.10	138	0.46	0.89
Decomposition ~ Diversity					
2014 – 2015	-0.010	0.003	138	-2.57	0.02
2014 – 2016	0.005	0.003	137	-1.58	0.25
2015 – 2016	0.004	0.003	138	1.34	0.37

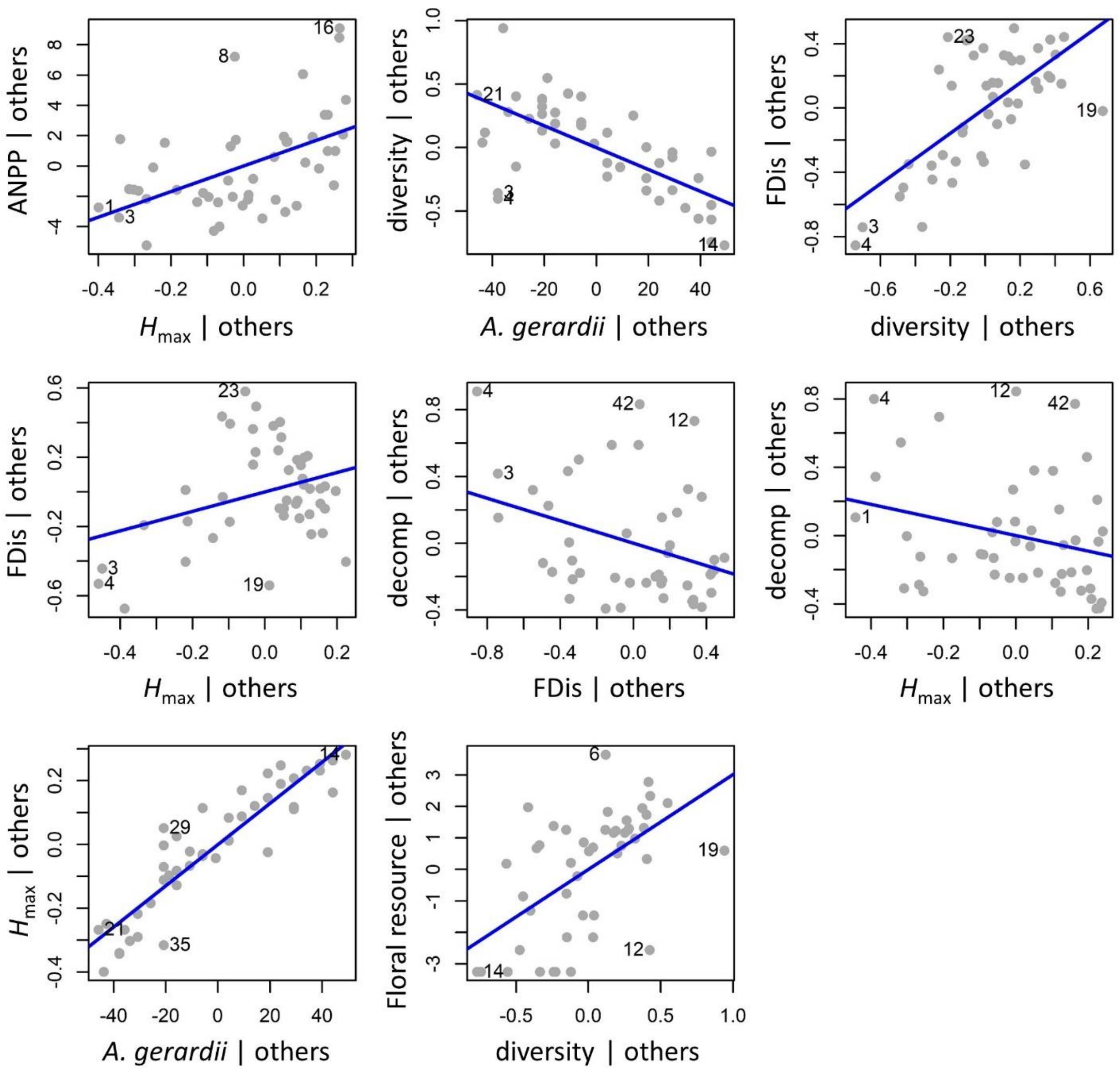

Figure S2 Partial regression plots of the paths summarized in the SEM for the 2014 establishment year communities (Fig 4a).

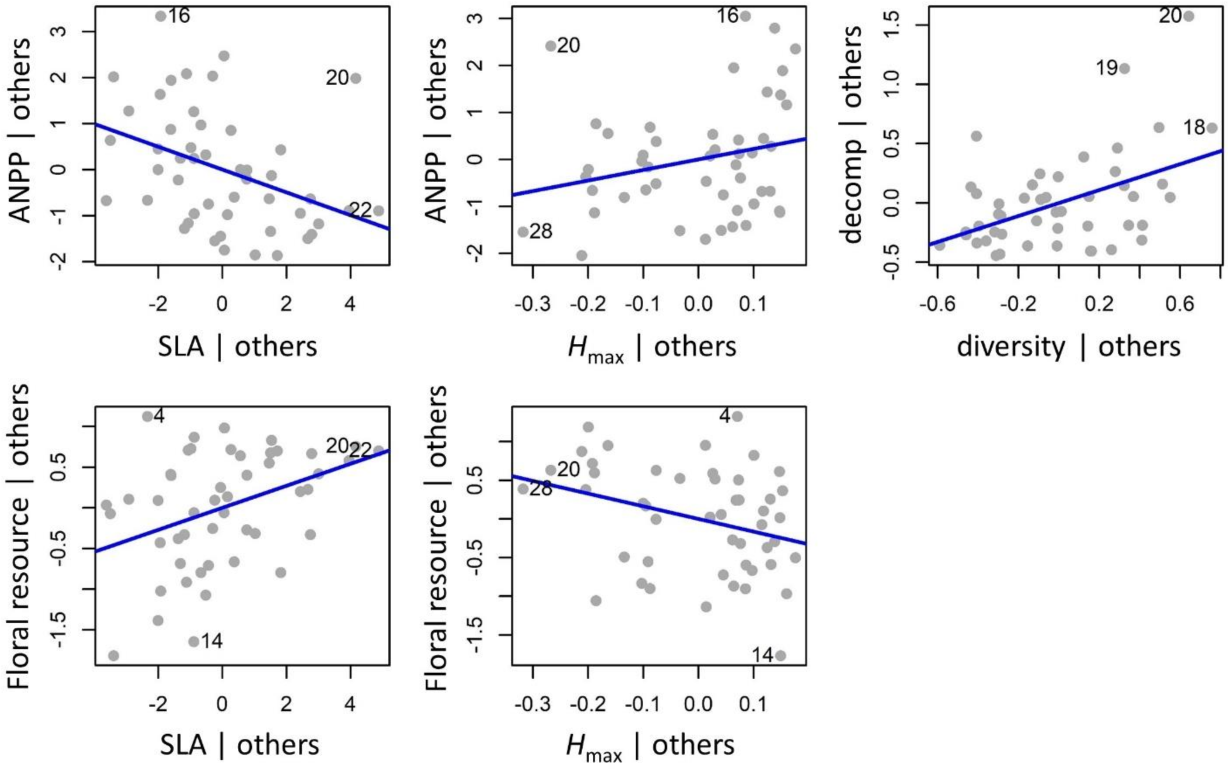

Figure S3 Partial regression plots of the paths summarized in the SEM for the 2015 establishment year communities (Fig. 4b).

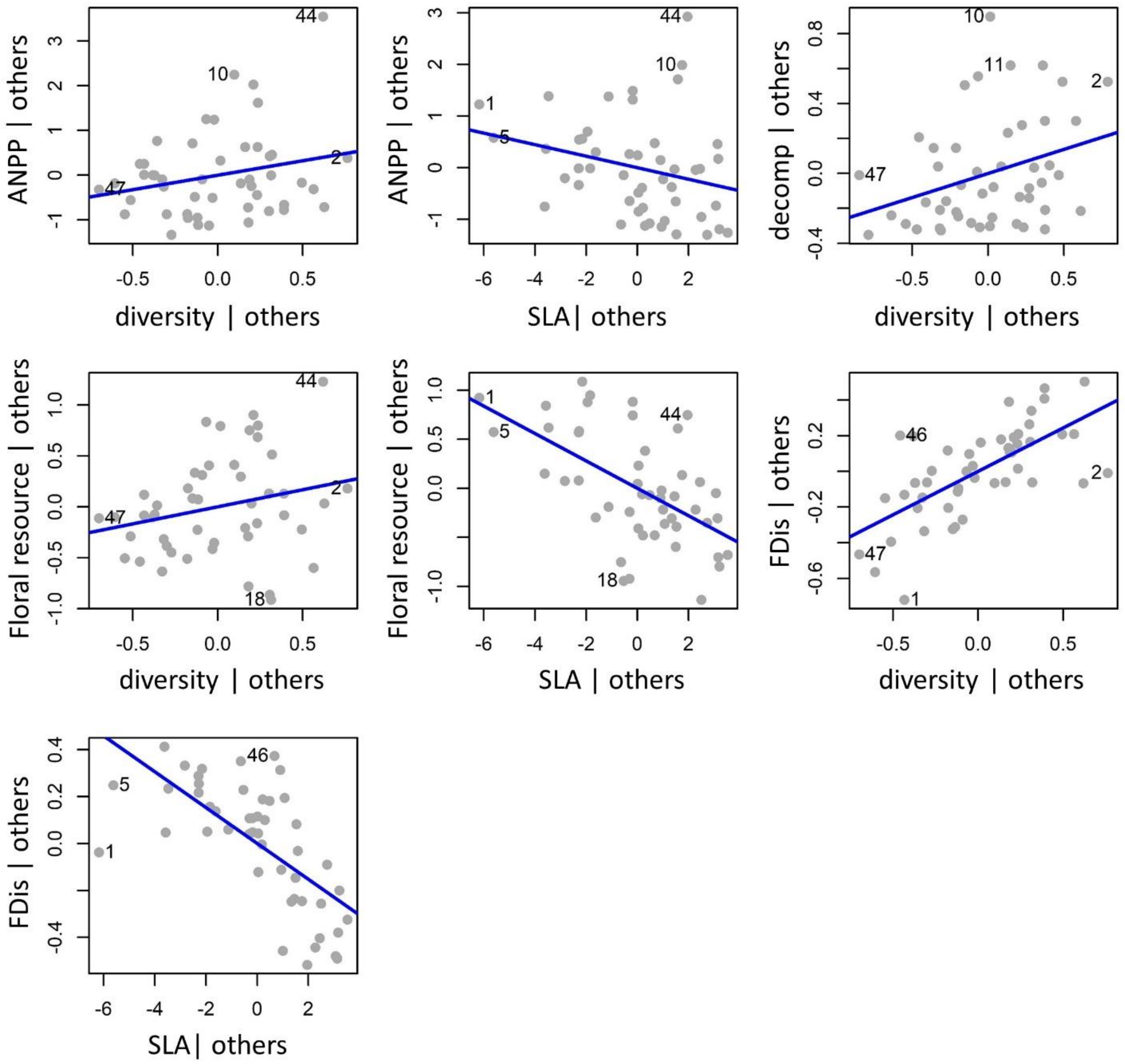

Figure S4 Partial regression plots of the paths summarized in the SEM for the 2016 establishment year communities (Fig. 4c).

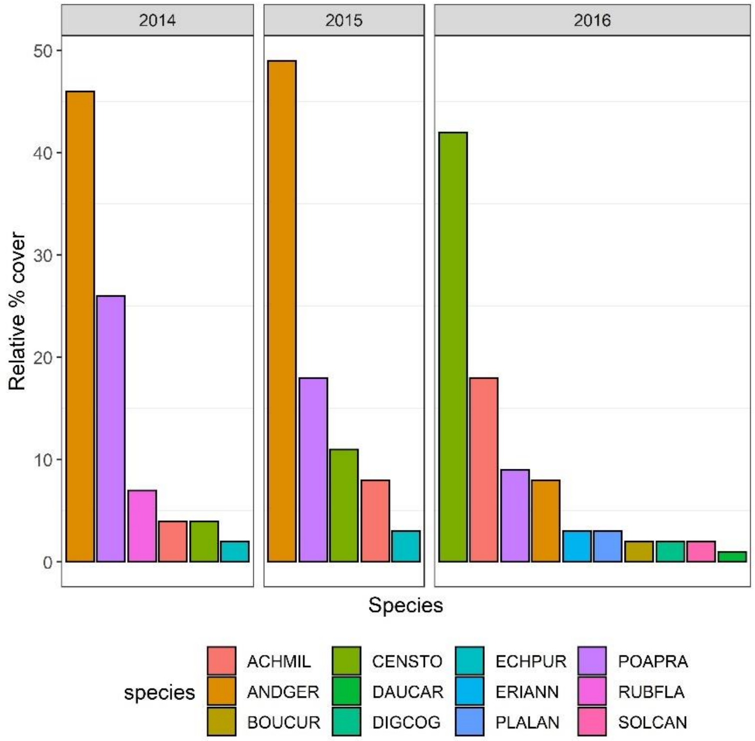

Figure S5 Rank-order plots of the species comprising 90% of the total percent cover in each establishment year.

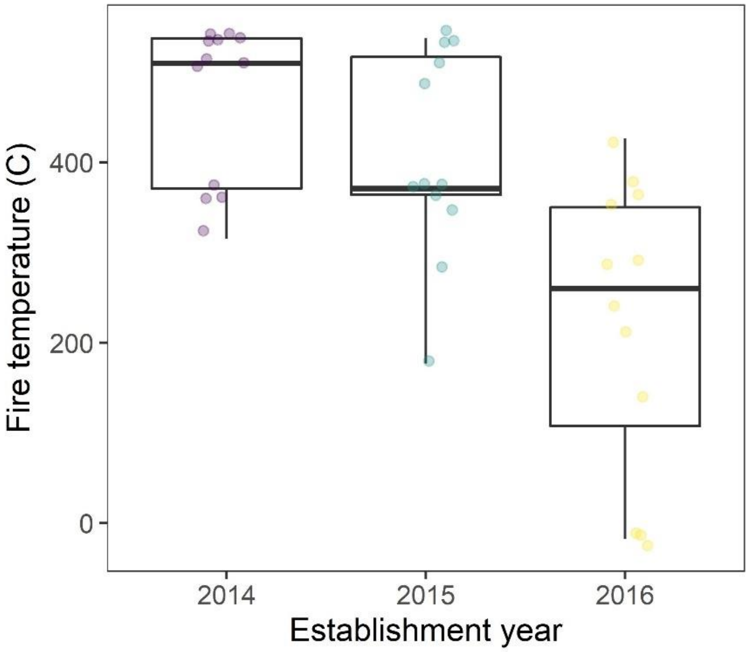

Figure S6 Boxplots showing fire temperature varied across the three establishment year treatments ($F_{2,15}$ = 11.09, P = 0.001) during a prescribed burn in April 2021. Fires in 2016 establishment year communities burned 252.3° C [95% CI: 107.4, 397] degrees cooler than fires in the 2014 establishment year communities, and 189.8° C [44.9, 335] degrees cooler than fires in the 2015 establishment year communities. Fire temperatures did not clearly differ between 2014 and 2015 establishment year communities (mean difference = 62.5° C [-82.4, 207]). Fire temperate was quantified using pyrometers (temperature-indicating paints streaked onto copper tags; paints indicated 150-450° C in 28° C increments), located at ground level adjacent to each 1×1m subplot sampled in 2020. Pyrometers were deployed immediately prior to the burn, retrieved immediately after, and read out by a single observer (LAB).

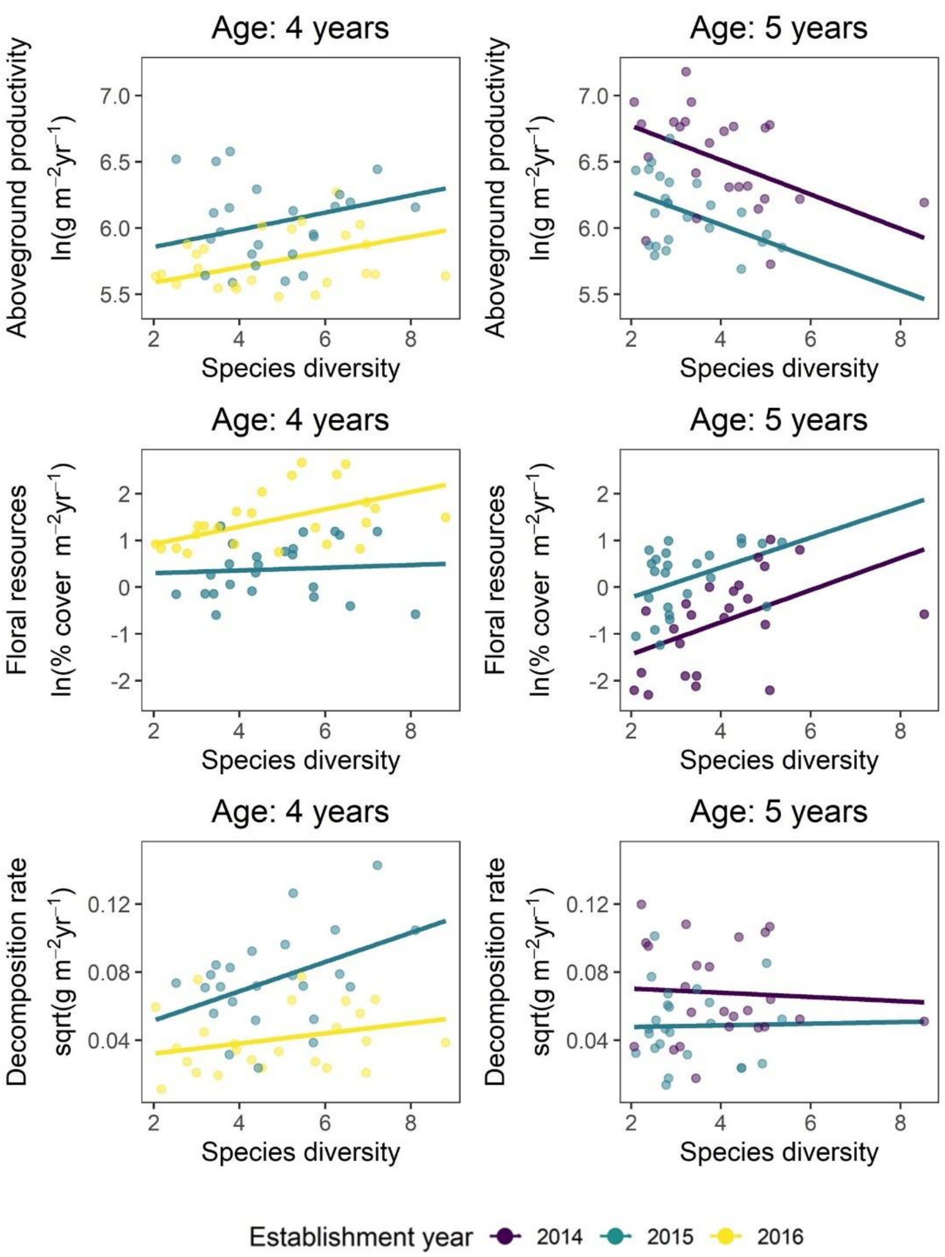

Figure S7 Differences in biodiversity-ecosystem functioning relationships in communities assembled from different establishment years, standardized by site age. Fitted lines are the predicted functions from mixed effects models (see Table S3).

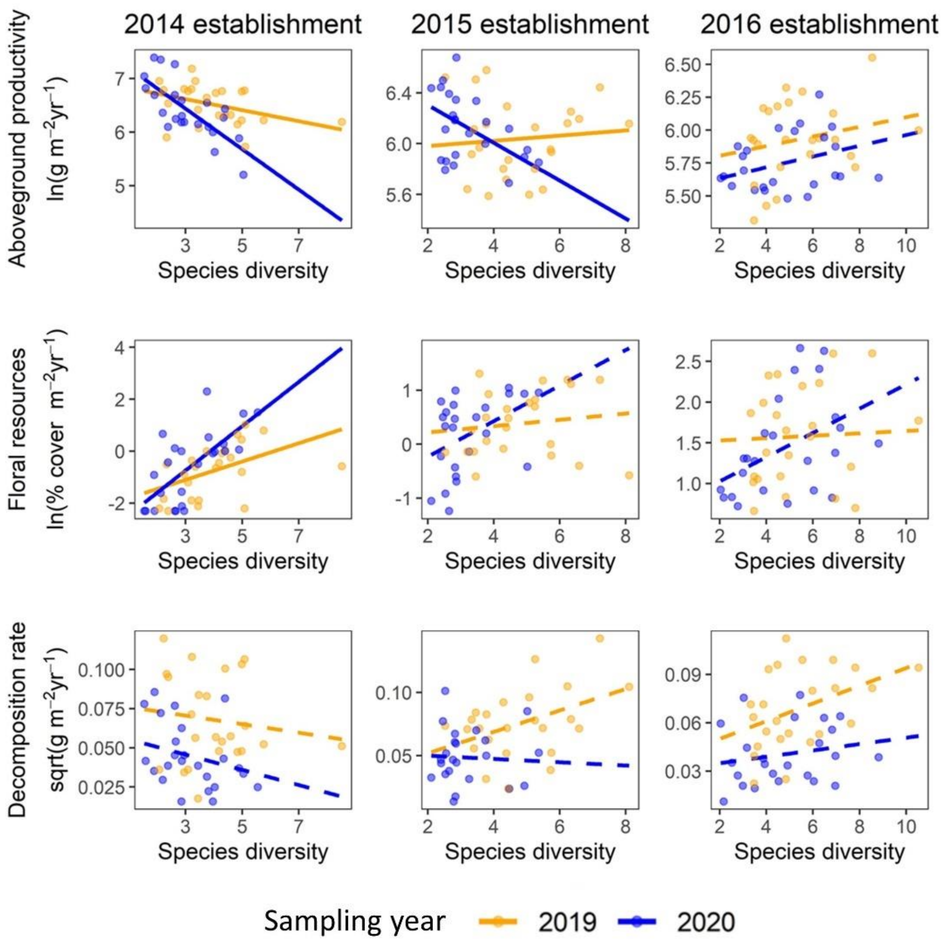

Figure S8 Differences in biodiversity-ecosystem functioning relationships over time (across sampling years) in each establishment year treatment. Fitted lines are the predicted functions from mixed effects models, where solid lines indicate slopes were statistically different and dotted lines indicate slopes were not statistically different (see Table S4).

Table S3: Pairwise comparison of mixed model slopes between species diversity and ecosystem functions (ANPP, Floral resource production, and decomposition rate) estimated in each establishment year at standardized site ages (see Fig S7). There were statistically clear establishment year effects ($p < 0.05$) for all functions when non-significant interaction terms were removed (except for decomposition at age 5). Degrees of freedom (df) were estimated with Kenward-Roger method. Estimates and SE are reported on the transformed scale of the response variable(s).

Establishment year contrast	estimate	SE	df	t ratio	p-value
Age 4 years (2015 − 2016)					
ANPP ~ Diversity	0.01	0.04	40.5	0.17	0.85
Floral resources ~ Diversity	-0.15	0.12	42.8	-1.31	0.19
Decomposition ~ Diversity	0.005	0.004	38.1	1.21	0.23
Age 5 years (2014 − 2015)					
ANPP ~ Diversity	-0.01	0.07	41.8	-0.07	0.94
Floral resources ~ Diversity	0.02	0.21	43.2	0.11	0.91
Decomposition ~ Diversity	-0.001	0.006	39.8	-0.27	0.78

Table S4: Mixed-model contrasts for differences in biodiversity-ecosystem functioning slopes across sampling years (time) in each establishment year treatment (see Fig S8). Degrees of freedom (df) were estimated with Kenward-Roger method. Estimates and SE are reported on the transformed scale of the response variable(s).

**Sampling year contrast**	**estimate**	**SE**	**df**	**t ratio**	**p-value**
2014 establishment year					
ANPP ~ Diversity	0.27	0.09	41.9	2.82	<0.01
Floral resources ~ Diversity	-0.50	0.24	43.6	-2.06	0.04
Decomposition ~ Diversity	0.002	0.005	41.2	0.40	0.68
2015 establishment year					
ANPP ~ Diversity	0.16	0.06	39.9	2.82	<0.01
Floral resources ~ Diversity	-0.27	0.16	41.5	-1.63	0.10
Decomposition ~ Diversity	0.009	0.006	42.6	1.51	0.13
2016 establishment year					
ANPP ~ Diversity	-0.00	0.03	41.6	-0.11	0.90
Floral resources ~ Diversity	-0.13	0.08	42.1	-1.55	0.12
Decomposition ~ Diversity	0.003	0.003	42.5	1.00	0.32
